# Supplementary material for: Multi-Modal Analysis of Satellite Cells Reveals Early Impairments at Pre-Contractile Stages of Myogenesis in Duchenne Muscular Dystrophy
Source: Cells. 2025 Jun 13;14(12):892. doi: 10.3390/cells14120892 (PMC12190492; doi:10.3390/cells14120892)
Supplement: Supplementary file 1 [file cells-14-00892-s001.zip › Supplementary_Figure_S1_NEW.pdf]

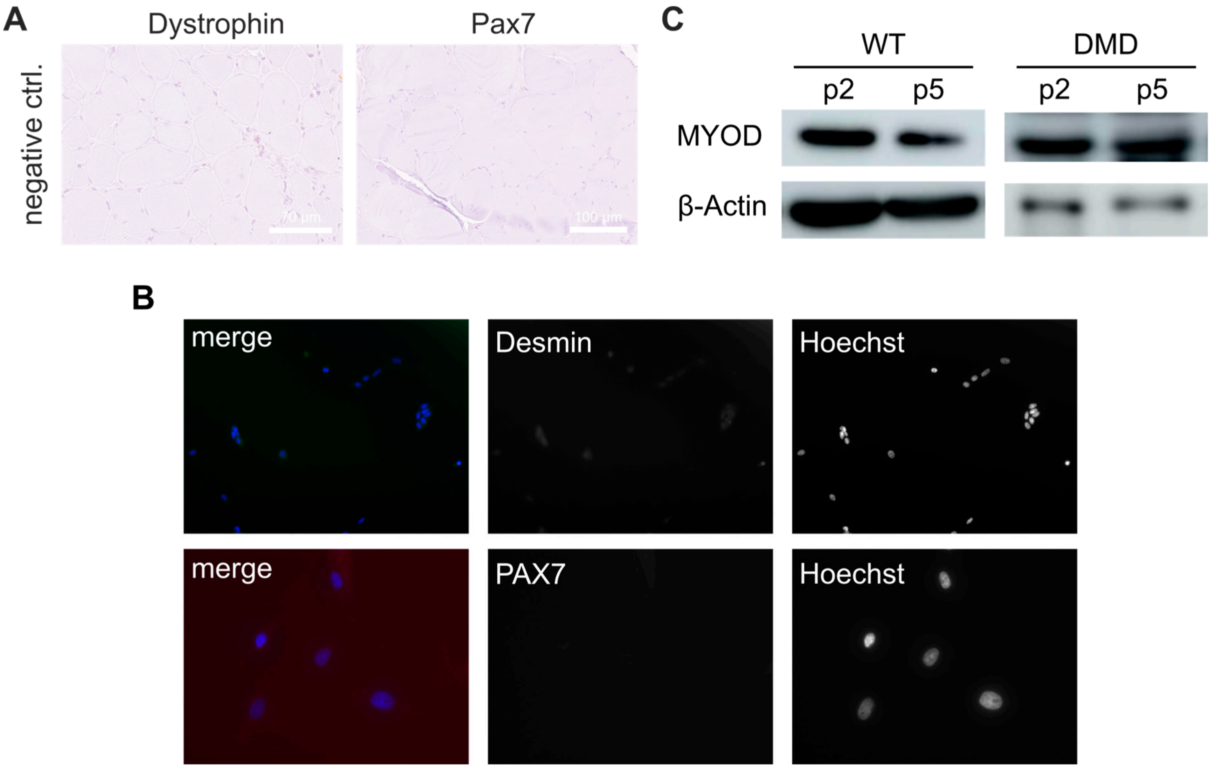

Figure S1: Negative controls for immunohistochemistry and immunofluorescence experiments and validation of myogenic nature of isolated SC

(A) *Negative controls (secondary antibody only) for immunohistochemistry of dystrophin and PAX7 in paraffin-embedded INT muscle section of DMD pigs. Scale bar = 100 µm and 70 µm.* ***(B)*** *Negative controls (secondary antibody only) for immunofluorescence staining of desmin (green) and PAX7 (red) of cultured DMD SC. Hoechst (blue) was used as a nuclear counterstain; magnification 20x for desmin and 40x for PAX7.* ***(C)*** *Western blot analysis to detect expression of myogenic marker MYOD in proliferating DMD and WT SC lysates; β-Actin was used as loading control.*
