# Supplementary material for: Multi-Modal Analysis of Satellite Cells Reveals Early Impairments at Pre-Contractile Stages of Myogenesis in Duchenne Muscular Dystrophy
Source: Cells. 2025 Jun 13;14(12):892. doi: 10.3390/cells14120892 (PMC12190492; doi:10.3390/cells14120892)
Supplement: Supplementary file 1 [file cells-14-00892-s001.zip › Supplementary_Figure_S2_NEW.pdf]

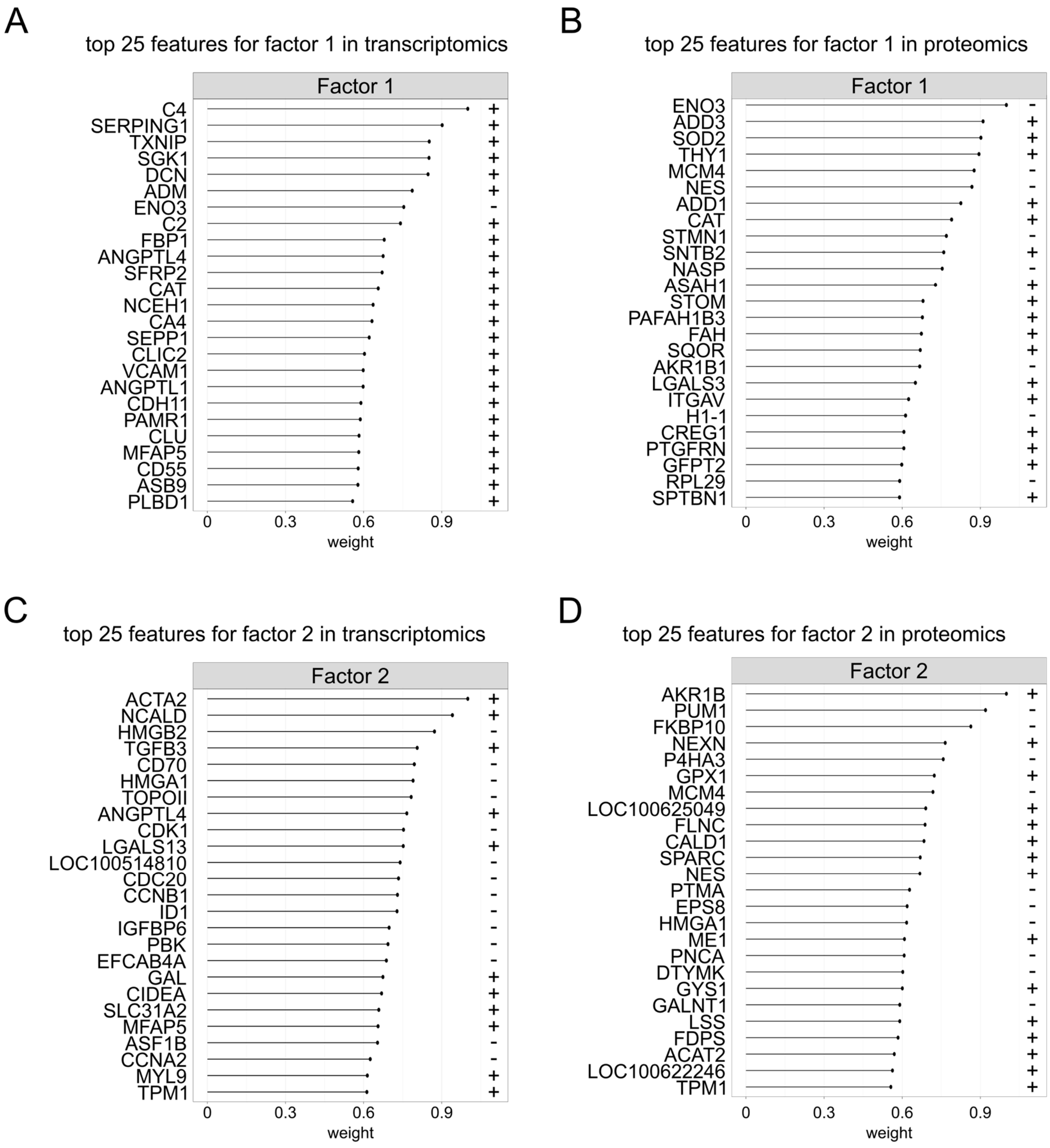

Figure S2: Top 25 features for factor 1 and 2 in transcriptomic and proteomic dataset and their sample expression

*Top 25 features for factor 1 in the transcriptomic **(A)** and proteomic **(B)** modality. Top 25 features for factor 1 in transcriptomic **(C)** and proteomic **(D)** modality. Features with large positive weights are more abundant in samples with a positive factor value, whereas features with a large negative weight are more abundant in samples with a negative factor value respectively.*
