# Supplementary material for: Multi-Modal Analysis of Satellite Cells Reveals Early Impairments at Pre-Contractile Stages of Myogenesis in Duchenne Muscular Dystrophy
Source: Cells. 2025 Jun 13;14(12):892. doi: 10.3390/cells14120892 (PMC12190492; doi:10.3390/cells14120892)
Supplement: Supplementary file 1 [file cells-14-00892-s001.zip › Supplementary_Method_S1.pdf]

Protocol for the isolation of porcine satellite cells from transgenic DMD and WT piglets

Material

Reagents and Resources

Chemicals		
Reagent	Source	Cat. Nr. / Identifier
Dulbecco's Phosphate-Buffered Saline (DPBS), no calcium, no magnesium	GIBCO Thermo Fisher	#14190-094
Dulbecco's Modified Eagle Medium (DMEM), high glucose, GlutaMAX^{TM} Supplement	GIBCO Thermo Fisher	10566-016
Ham's F10 Nutrient Mix,	GIBCO Thermo Fisher	31550-023
Fetal Bovine Serum (FBS)	GIBCO Thermo Fisher	10270-106
Penicillin-Streptomycin (10.000 u/mL),	GIBCO, Thermo Fisher	15140-122
HEPES buffer solution	Sigma Aldrich	83264
Recombinant Human FGF-basic (154 a.a.)	PEPro Tech	100-18B
Basalmembranematrix, Corning® Matrigel	VWR	734-0270
Protease from *Streptomyces griseus*	Sigma Aldrich	P8811
Collagenase from *Clostridium histolyticum*	Sigma Aldrich	C9407
autoMACS Running Buffer	Miltenyi Biotec	130-091-221
Ethanol 70%		

Antibodies		
Reagent	Source	Cat. Nr. / Identifier
Anti-CD31	Abcam	ab28364
Anti-CD45	Abcam	ab10558
Anti-Integrin-β1,	Abcam	ab30388
Anti-NCAM1	Abcam	ab9018
Anti-M-cadherin	Santa Cruz	sc-374093
Anti-Rabbit IgG MicroBeads	Miltenyi Biotec	130-048-602
Anti-Mouse IgG MicroBeads	Miltenyi Biotec	130-047-102

Equipment		
Reagent	Source	Cat. Nr. / Identifier
Sterile laminar flow hood	Thermo Scientific	Maxisafe 2030i Biosafety Cabinet
CO2 Incubator	Thermo Scientific	HERA cell 150i
Table top centrifuge	Sigma Laboratory Centrifuges	4K15
Microcentrifuge	Eppendorf	5417 R,
Scalpel blades	Heinz Herenz	0482
Centrifuge tube, 15 mL	Greiner	188271
Centrifuge tube 50 mL	Greiner	227261
Reaction tube, 1.5 mL	Greiner	616201
Reaction tube, 2.0 mL	Greiner	623201
Cell culture multiwall plate,	Greiner	665180
Cell culture dish, 100/20 mm,	Greiner	664160
EasyStrainer 40 µm,	Greiner	542040
EasyStrainer 70 µm,	Greiner	542070
MACS separator	Miltenyi Biotec	-
LS column	Miltenyi Biotec	130-042-401
MS column	Miltenyi Biotec	130-042-201

Solutions

Transport medium		
Reagent	Final Concentration	Amount
DMEM	-	39.5 mL
FBS	20 %	10 mL
Penicillin-Streptomycin	1%	0.5 mL
total		50 mL

Isolation medium (IsM)		
Reagent	Final Concentration	Amount
DMEM	-	44.5 mL
FBS	10 %	5 mL
Penicillin-Streptomycin	1 %	0.5 mL
total		50 mL

Washing buffer (PBS+)		
Reagent	Final Concentration	Amount
DPBS	-	49.5 mL
Penicillin-Streptomycin	1 %	0.5 mL
total		50 mL

Protease digestion		
Reagent	Final Concentration	Amount
DPBS	-	9 mL
Protease	1.5 mg/mL	15 mg
HEPES buffer	1 %	0.5 mL
Penicillin-Streptomycin	1 %	0.5. mL
total		10 mL

Collagenase digestion		
Reagent	Final Concentration	Amount
DMEM	-	9.5 mL
Collagenase	1.5 mg/mL	15 mg
FBS	5 %	0.5 mL
total		10 mL

Growth medium for purified satellite cells (GM)		
Reagent	Final Concentration	Amount
Ham's F10		395 mL
FBS	20 %	100 mL
0.1 µg/µL bFGF	0.0002%	100 µL
Pencillin-Streptomycin	1 %	5 mL
total		500 mL

Workflow

1. Working under a sterile lamina flow: transfer muscle biopsies from transport tubes into a 10-cm dish
2. Remove attaching connective tissue and mince the samples using a scalpel blade
3. Sterilize tissue using 70% ethanol
4. Rinse with cold PBS+
5. Transfer the tissue in a 50 mL tube and add ~1 mL PBS+ with 1% HEPES
6. Centrifuge for 5 min at 300 g
7. Transfer the supernatant (SN1) into a 15 mL tube and store on ice until further usage
8. To the remaining cell pellet, add ~5 mL Protease digestion solution (tissue completely covered)
9. Incubate at 37°C for 1 h, shake the tube by hand every 10-15 min
10. Centrifuge for 5 min at 300 g
11. Add the supernatant to SN1 and store on ice until further usage (SN2)

12. To the pellet, add ~5 mL Collagenase digestion solution (tissue completely covered)
13. Incubate at 37°C for 1 h, shake the tube by hand every 10-15 min
14. During the incubation time, continue working with SN2:
    14..1.Centrifuge for 5 min at 300 g
    14..2.Aspirate the supernatant and keep the "pellet"
    14..3.Resuspend the pellet in 1 mL IsM
    14..4.Filter through 70 µm filter into a 15 mL tube (SN3) and store on ice until further usage
15. Remove the tube from the incubator and sift through 70 µm filter into 50 mL tube
16. Centrifuge for 10 min at 300 g
17. Aspirate the supernatant
18. Resuspend the pellet with SN3
19. Sift through 40 µm filter into 50 mL tube
20. For washing, add 5 mL PBS+ and centrifuge for 5 min at 300 g
21. Aspirate the supernatant and add 5 mL IsM
22. Centrifuge for 10 min at 700 g
23. Aspirate the supernatant, resuspend the pellet in 200 µL IsM and transfer the cell solution into a 1.5 mL tube
24. Add 1 µl of each antibody to the suspension: anti-CD31, anti-CD45, anti-Integrin-β1, anti-NCAM1, anti-M-cadherin
25. Incubate on ice for 30 min
26. Wash twice: add 1 mL IsM, centrifuge for 3 min at 300 g at 4°C, aspirate supernatant
27. Resuspend the pellet in 200 µL IsM
28. Add 15 µL anti-Rabbit IgG MicroBeads
29. Incubate on ice for 30 min
30. Wash twice: centrifuge for 3 min at 300 g at 4°C, aspirate supernatant, resuspend in 1 mL MACS buffer
31. Set up a LS column on the MACS separator and place a 15 mL tuber underneath the column to collect the flow-through, pre-balance the column by rinsing with MACS buffer
32. Resuspend the cell pellet in 1 mL MACS buffer and transfer to the LS column
33. Gently insert the plunger and sift the solution through the column
34. Continue working with the flow-through
35. Centrifuge for 3 min at 300 g at 4°C
36. Aspirate the supernatant and resuspend the pellet in 200 µL IsM
37. Add 15 µL anti-Mouse IgG MicroBeads
38. Incubate on ice for 30 min

39. Wash twice: centrifuge for 3 min at 300 g at 4°C, aspirate supernatant, resuspend in 1 mL MACS buffer
40. Set up a MS column on the MACS separator and place a 15 mL tuber underneath the column to collect the flow-through, pre-balance the column by rinsing with MACS buffer
41. Resuspend the cell pellet in 1 mL MACS buffer and transfer to the MS column
42. Gently insert the plunger and sift the solution through the column
43. Remove the flow-through
44. Place a new 15 mL tube underneath the column and remove tube and column from the magnetic field together
45. Elute the cells by flushing the tube with 1 mL MACS buffer, repeat twice
46. Centrifuge for 3 min at 300 g at 4°C
47. Aspirate the supernatant and resuspend in 1 mL GM
48. Count the cells with a Neubauer Chamber
49. Seed the cells directly on a 10% Matrigel coated 12-well plate and incubate at 37°C
